# Characterization of 17β-estradiol-degrading enzyme from *Microbacterium* sp. MZT7 and its function on E2 biodegradation in wastewater

**DOI:** 10.1186/s12934-023-02119-w

**Published:** 2023-06-27

**Authors:** Peng Hao, Hanyu Pan, Zongshuo Lv, Jingyi Zhang, Lixia Wang, Yanbin Zhu, Wangdui Basang, Yunhang Gao

**Affiliations:** 1 College of Veterinary Medicine, Jilin Agricultural University, Changchun, 130118 China; 2 Institute of Animal Husbandry and Veterinary Medicine, Tibet Academy of Agriculture and Animal Husbandry Science, Lhasa, 850009 China; 3 Northeast Institute of Geography and Agroecology, Chinese Academy of Sciences, Changchun, 130102 China

**Keywords:** 17β-estradiol, Enzyme, Remediation, Wastewater, *Microbacterium* sp. MZT7

## Abstract

**Background:**

17β-estradiol (E2) residues exhibit harmful effects both for human and animals and have got global attention of the scientific community. Microbial enzymes are considered as one of the effective strategies having great potential for removal E2 residues from the environment. However, limited literature is available on the removal of E2 from wastewater using short-chain dehydrogenase.

**Results:**

In this study, 17β-estradiol degrading enzyme (17β-HSD-0095) was expressed and purified from *Microbacterium* sp. MZT7. The optimal pH and temperature for reaction was 7 and 40 °C, respectively. Molecular docking studies have shown that the ARG215 residue form a hydrogen bond with oxygen atom of the substrate E2. Likewise, the point mutation results have revealed that the ARG215 residue play an important role in the E2 degradation by 17β-HSD-0095. In addition, 17β-HSD-0095 could remediate E2 contamination in synthetic livestock wastewater.

**Conclusions:**

These findings offer some fresh perspectives on the molecular process of E2 degradation and the creation of enzyme preparations that can degrade E2.

**Graphical Abstract:**

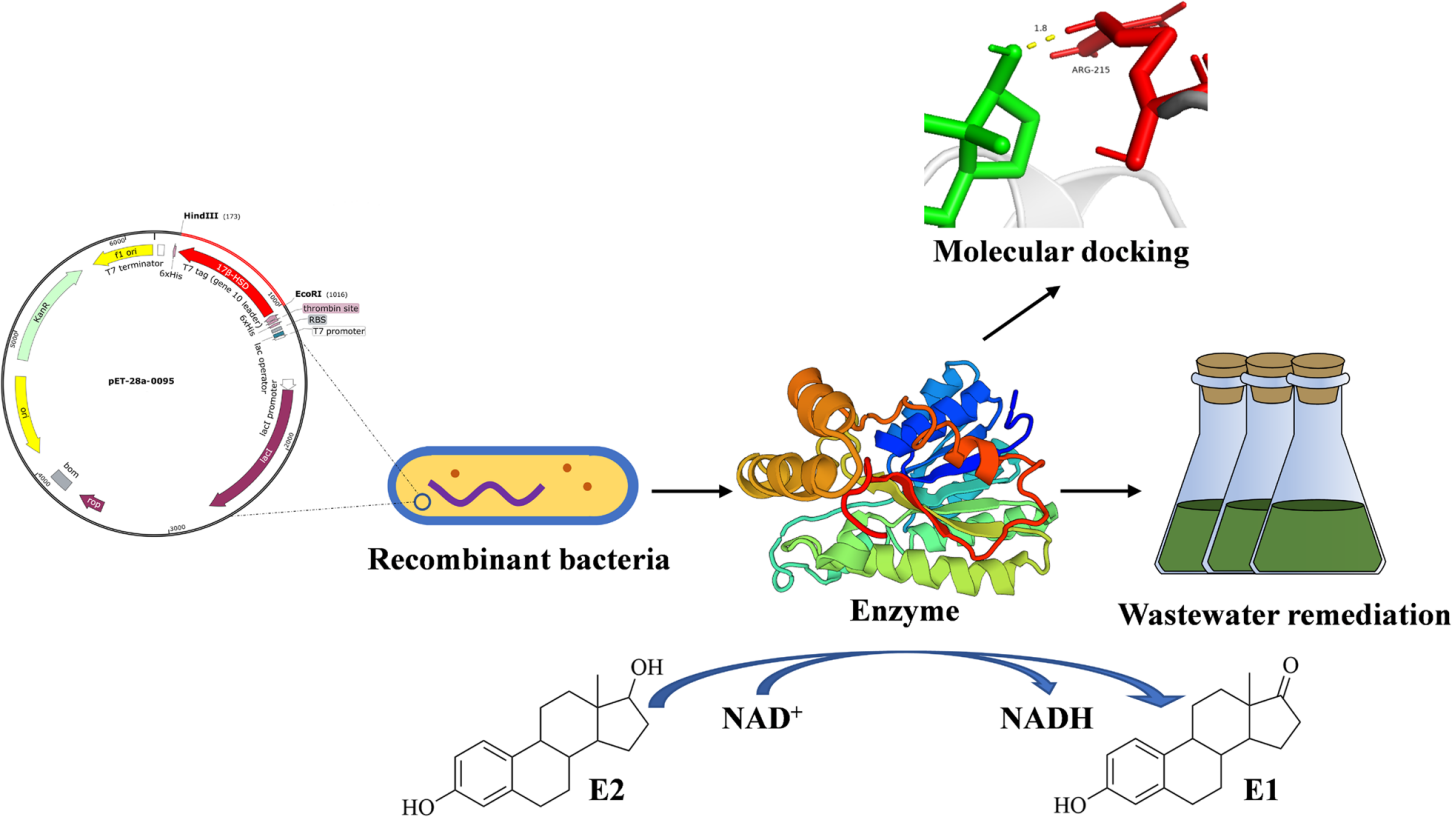

## Introduction

Natural steroidal estrogens (SEs) are contaminants mainly produced by humans and animals and excreted into the environment through urine and feces [1, 2]. With the development of human activities and livestock farming, endocrine-disrupting compounds could be easily detected in various water systems [3]. The review article by Tremblay et al. has reported the presence of steroidal estrogens in agricultural fertilizers and wastewater at concentrations ranging from 40–7000 ng/L [4]. Scientific investigations have shown that they have adverse effects on the ecological and biological health [5, 6]. Strong biological activity has been observed even at the level [7, 8]. Estradiol (E2) is a typical member of the estrogen family and has the highest estrogenic activity. This is the most potent form of estrogen, and its concentration is highest in females of childbearing age. Some physicians prescribe it as a marker for ovary health [9, 10]. The levels of 17β-estradiol in rivers and freshwater have been reported by wastewater treatment plants (WWTPs) in North America to be 1–22 ng/L and 0–4.5 ng/L, respectively [11]. Long-term exposure to an environment contaminated with E2 can affect the health of organisms, resulting in feminization of male fish, stunted plant growth and development, reduced fertility, decreased sperm count and increased risk factors for cancer etc. [1, 5, 12–15]. Now, E2 has been categorized as a Group 1 carcinogen by the World Health Organization [16]. Therefore, bioremediation of estrogens in the environment is crucial.

Functional microbial degradation of E2 is a green and cost-effective method [17, 18]. Currently, many E2-degrading bacteria have been isolated, which can convert E2 to substances with low or no estrogenic activity using estrogen as the only carbon source substance [7, 19, 20]. It is well known that the E2 biodegradation occurs mainly through enzyme catalysis. In most of the reported E2-degrading bacteria, enzyme-catalyzed conversion of E2 to estrone (E1) is considered as the first reaction step in E2 degradation [7, 20–22]. This step of reaction is the key and restriction step for E2 to be biodegraded [23]. According to the investigations, the estrogenic potency of E2 is much greater than that of E1 [24, 25]. This implies that E2 is more environmentally toxic than E1 [26]. Therefore, it is of great importance to explore the degrading enzymes that enable the biodegradation of E2 to E1. Hydroxysteroid dehydrogenases (HSDs) of the short-chain dehydrogenase/reductase (SDR) family are involved in redox at the E2 C17 position [7, 20]. Although many degrading bacteria have been reported, but there have been very few studies on the characterization and application of degrading enzymes.

The SDR belongs to the NAD(P)(H)-dependent oxidoreductase superfamily [27, 28]. The motif (Gly-X-X-X-Gly-X-Gly) involved in cofactor binding and the active site motif (Tyr-X-X-X-Lys) are two motifs shared by the SDR family members, in which Tyr, Lys and conserved Ser residues constitute a catalytic triad [29–31]. Despite the low sequence identity (only 15–30%) among SDR family members, their tertiary structures are strikingly consistent. That is, the coenzyme-binding domains with NAD(P)(H)-binding is *βαβ* motifs containing the conserved Rossmann fold [29]. Studies have shown that SDR can participate in the metabolism of a variety of substances [27, 32, 33].

*Microbacterium resistens* MZT7 (*M. resistens* MZT7) was isolated from dairy farm activated sludge and was subjected to whole genome sequencing (Accession number CP082781) [22]. Previous studies have shown that strain MZT7 can degrade E2 to E1 [22]. In another recent study, a heterologously expressed short-chain dehydrogenase gene (MZT7_GM000095, locus_tag = K8F61_00480) was confirmed to transform E2 to E1 [34]. In this work, an E2-degrading enzyme was expressed from strain MZT7 and characterized by biochemical characterization experiments. In addition, its mechanism of action was investigated by sequence analysis, homology modeling, molecular docking and point mutation. Finally, the ability of enzyme to remediate E2 in synthetic livestock wastewater was investigated.

## Results

### Sequence analysis

**Fig. 1:**
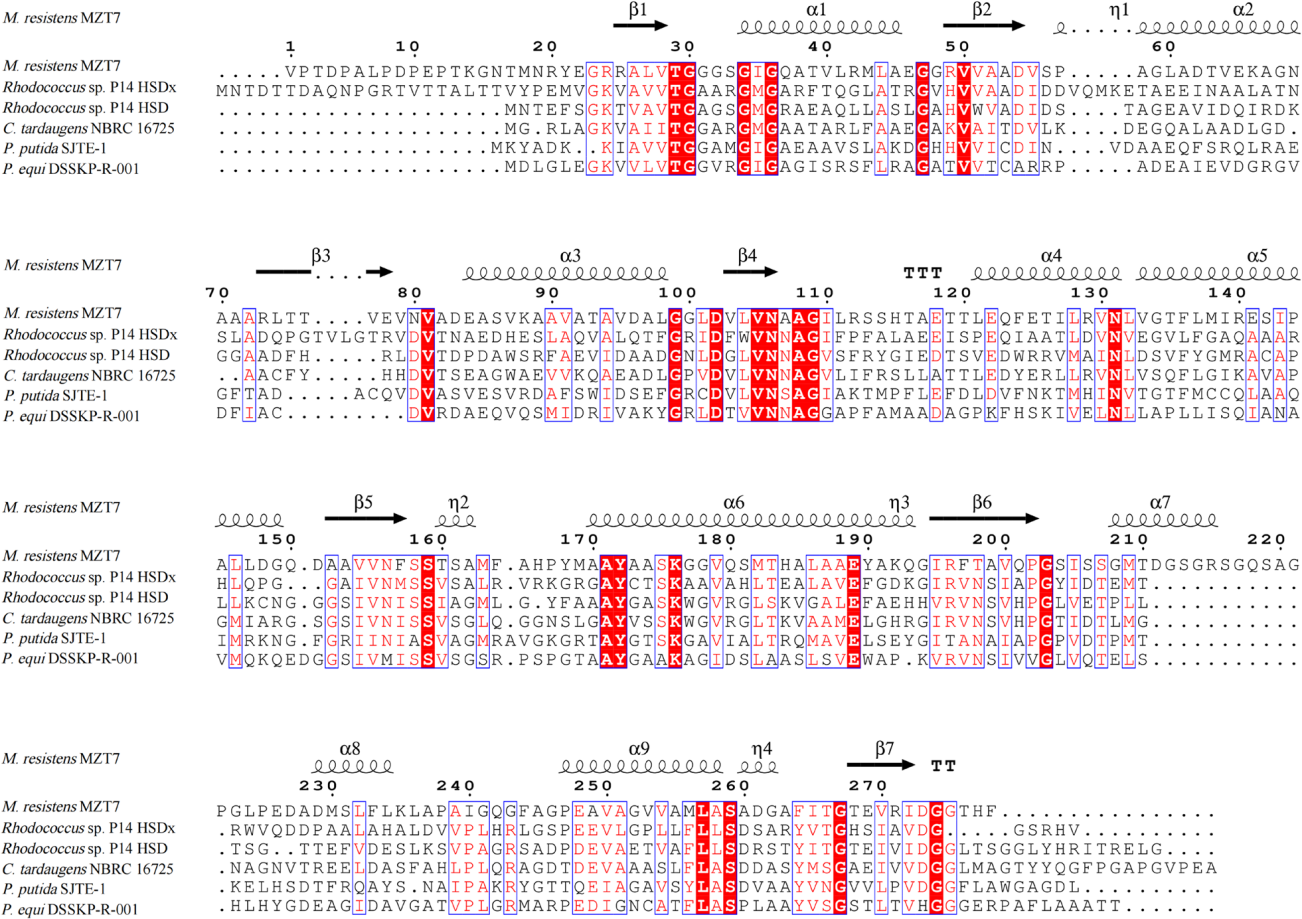
Alignment of 17β-HSD-0095 sequence with other 17β-HSD sequences. *M. resistens* MZT7 (UGS26750.2); *Rhodococcus* sp. P14 17β-HSDx (WP_010595925.1); *Rhodococcus* sp. P14 17β-HSD (ASC49558.1); *C. tardaugens* NBRC 16725 (AZI34912.1); *P. putida* SJTE-1 (ANI02794.1); *P. equi* DSSKP-R-001 (WP_005515558.1)

The genome and transcriptome of strain MZT7 were analyzed. A gene (*17β-HSD-0095*, locus_tag = K8F61_00480) with up-regulated expression under E2 pressure encoded HSD protein. The length of *17β-HSD-0095* gene was 837 bp, encoding a 278-amino acid protien (UGS26750.2) with a theoretical molecular weight of 28.03 kDa. Reported protein sequences with E2 degradation functions were selected for comparison of conservatism (Fig. 1). The results of multiple sequence comparison showed that 17β-HSD-0095 protein also has two conserved sequence motifs identical to other SDR family members: the NADH binding region (Gly30-X-X-X-Gly34-X-Gly36) and the acceptor proton region (Tyr172-X-X-X-Lys176). In addition, the sequence similarities between the 17β-HSD-0095 protein and other 17β-HSD proteins were 25.60% (WP_010595925.1), 20.86% (ASC49558.1), 27.30% (AZI34912.1), 22.22% (ANI02794.1) and 21.15% (WP_005515558.1), respectively.

### Expression and purification of 17β-HSD-0095

**Fig. 2:**
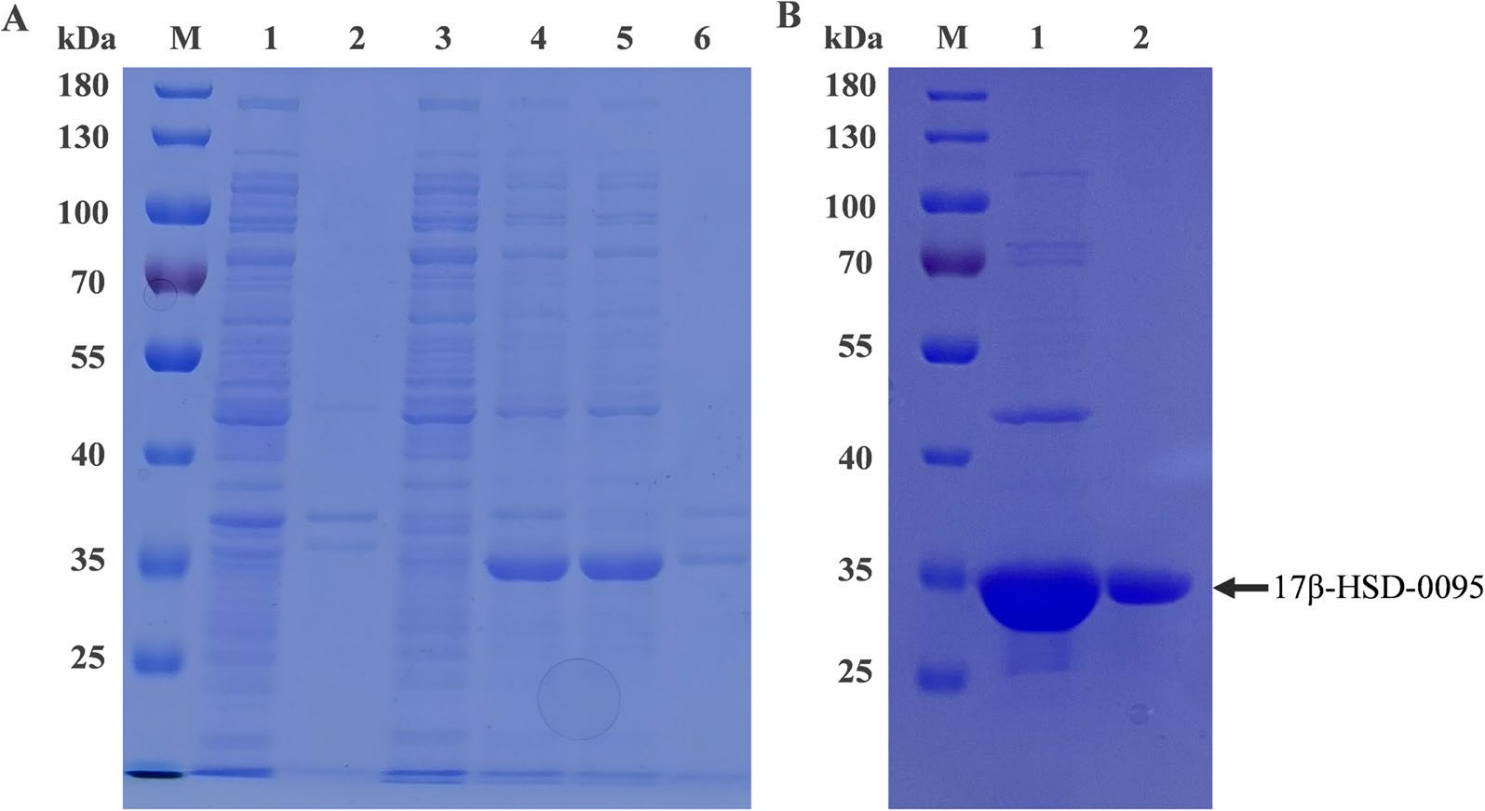
SDS-PAGE analysis revealed the expression and purification of 17β-HSD-0095 protein. **A** Lane M: molecular standard marker; Lanes 1–3: recombinant *E. coli* cells disruption solution without IPTG induction, supernatant of disruption solution and precipitate, respectively, Lanes 4–6: recombinant *E. coli* cell disruption solution induced by IPTG, supernatant of disruption solution and precipitate, respectively. **B** Lane M: molecular standard marker; Lane 1: purified protein eluate; Lane 2: purified protein eluate

*Escherichia coli* (BL21) carrying the recombinant plasmid was induced by IPTG to express the target protein (Fig. 2). 17β-HSD-0095 protein was shown to be expressed in soluble form via SDS-PAGE analysis (Fig. 2A). In addition, the molecular weight of the 17β-HSD-0095 protein with 6 × His tags determined by SDS-PAGE was approximately 35 kDa, which was consistent with the theoretical molecular weight (Fig. 2B). About 30 mg enzyme was purified from 1 L of culture.

### Characterization of 17β-HSD-0095

We investigated the effect of different conditions on the enzymatic activity of 17β-HSD-0095.

**Fig. 3:**
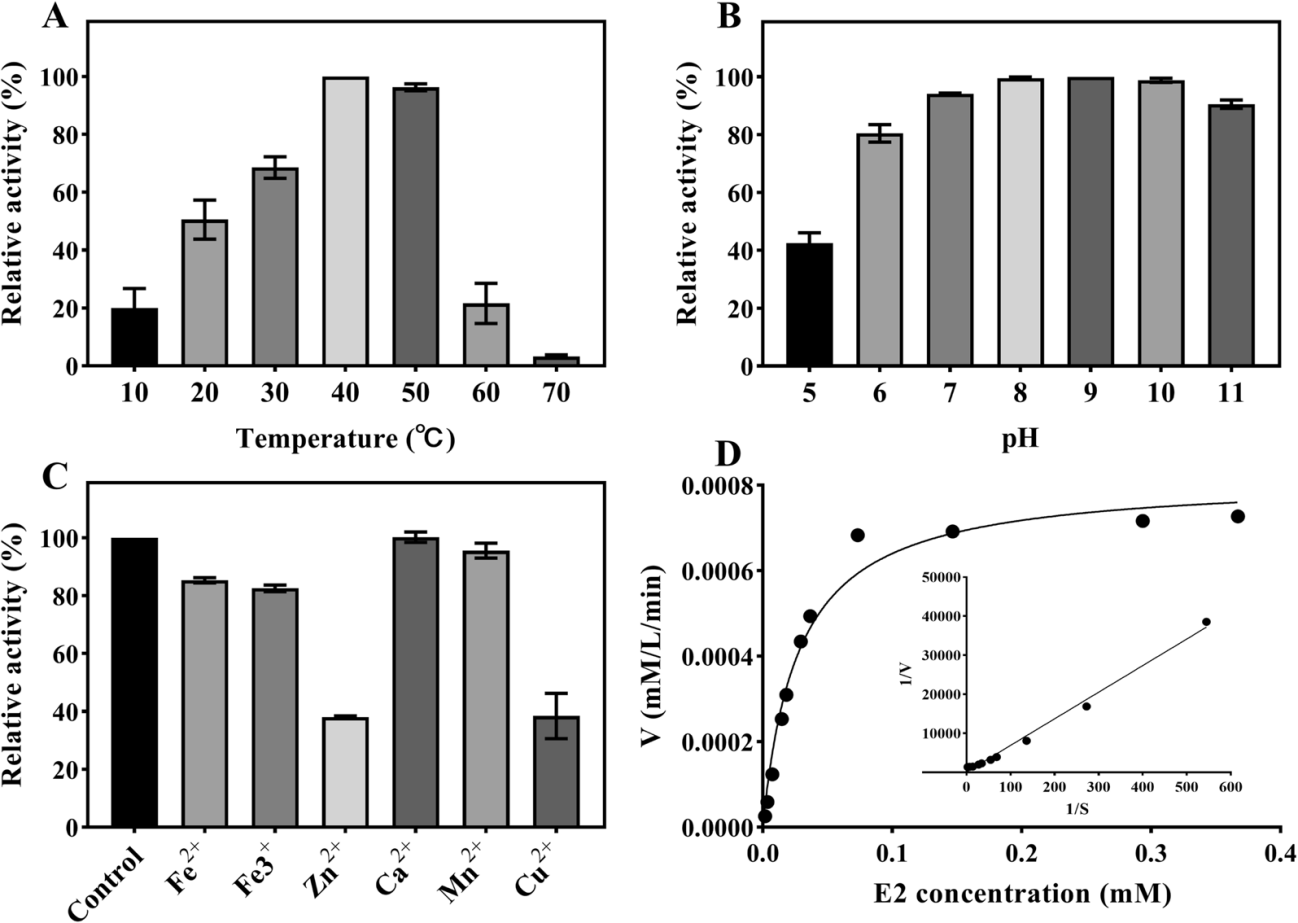
Characterization of protein 17β-HSD-0095. **A** Effect of temperature on the enzymatic activity of 17β-HSD-0095. **B** Effect of pH on the enzymatic activity of 17β-HSD-0095. **C** Effect of 1 mM of metal ions on the enzymatic activity of 17β-HSD-0095. **D** Kinetic profile of 17β-HSD-0095

The enzymatic activity of 17β-HSD-0095 was the highest (100) at 40 °C and decreased to the level 20 when the temperature exceeded 50 °C, and the degradative enzyme was inactivated when the temperature reached 70 °C (Fig. 3A). The enzymatic activity was measured in MSM solution at pH 5–11, and the results showed that 17β-HSD-0095 had higher enzymatic activity in alkaline conditions than in acidic conditions, and had the optimum enzymatic activity at pH 9 (Fig. 3B). The effects of different metal ions (Fe^2+^, Fe^3+^, Zn^2+^, Ca^2+^, Mn^2+^ and Cu^2+^) on enzymatic activity 17β-HSD-0095 are shown in Fig. 3C. All ions except Ca^2+^ and Mn^2+^ significantly inhibited (*p* < 0.05) enzymatic activity, with Zn^2+^ having the strongest inhibitory effect, reducing the enzymatic activity by 62%. The *Km* value of the recombinant enzyme was 0.48 mM and the *Vmax* value was 7.06 μM/min when the substrate was E2 (Fig. 3D).

### Homology modeling and molecular docking of 17β-HSD-0095

**Fig. 4:**
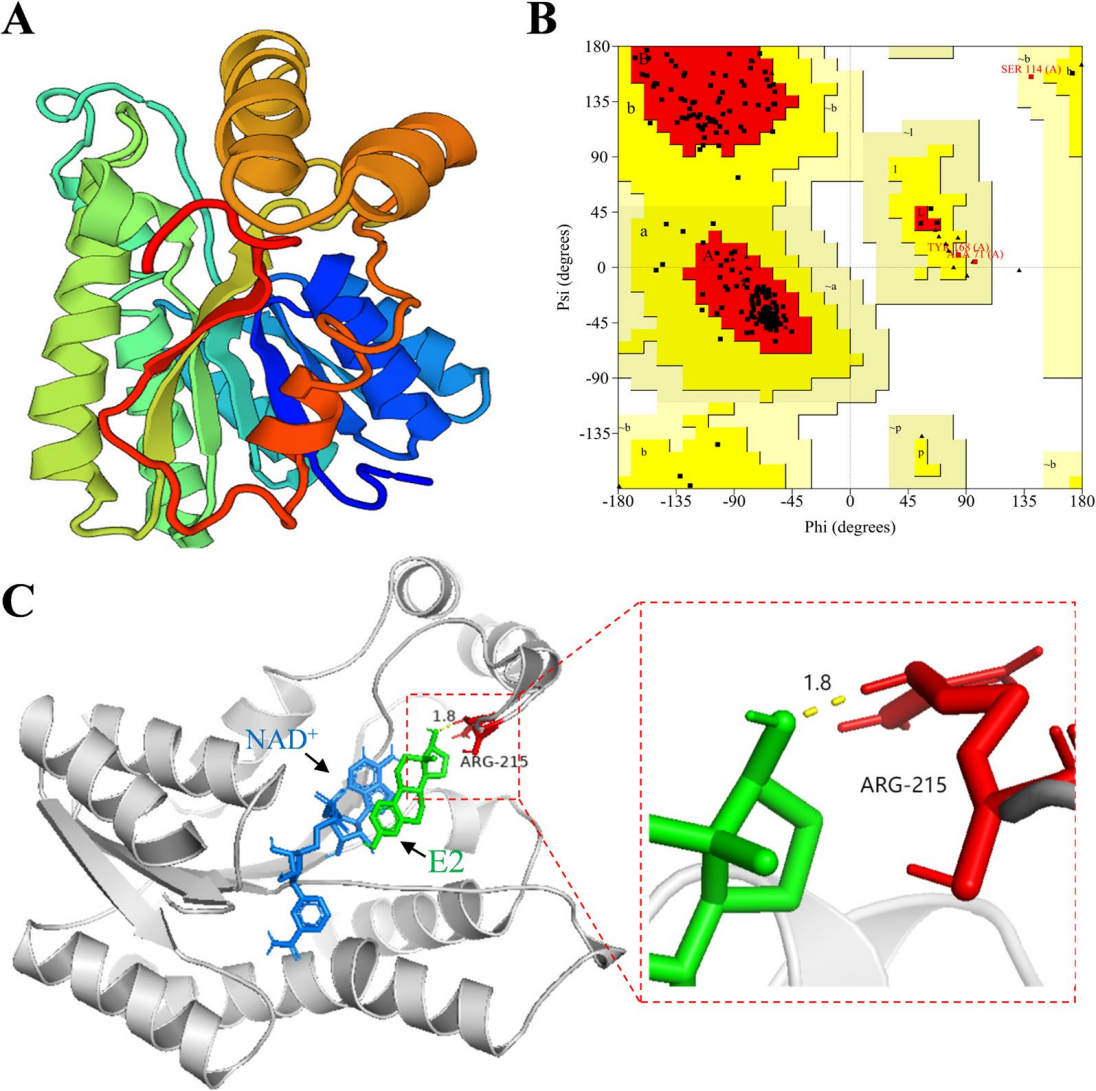
Homology modeling and molecular docking of 17β-HSD-0095. **A** 3-D structure of 17β-HSD-0095 predicted by SWISS-MODEL server. **B** Ramachandran plot of the 17β-HSD-0095. **C** Binding pattern of E2 with 17β-HSD-0095. Blue stick represents NAD^+^, green stick represents E2, red stick represents amino acid residues, and yellow dashed line represents hydrogen bond

To further investigate the mechanism of E2 degradation by 17β-HSD-0095, we predicted the 3-D structure of 17β-HSD-0095 by homology modeling (Fig. 4A). 17β-HSD-0095 showed 33.73% sequence identity with the template (PDB code: 8CXA). Ramachandran map results showed 90.3% amino acids (196) in the most favored regions, 8.3% amino acids (19) in the additional favored regions and no amino acids in the disallowed regions, indicating that the predicted model structure was reliable (Fig. 4B). The protein (17β-HSD-0095) and ligand (E2) were preprocessed using AutoDockTools prior to molecular docking. Then, docking was performed and the conformation with the lowest binding energy (− 8.31 kcal/mol) was selected for analysis. The correlation pattern maps of key amino acids to substrate binding sites were visualized by PyMOL (Fig. 4C). The results showed that the amino acid residue ARG215 of 17β-HSD-0095 and the oxygen atom of E2 (on the hydroxyl group at C17 of E2) formed a hydrogen bond with a hydrogen bond length of 1.8 Å.

### Effect of wild-type and mutant proteins on E2 degradation

**Fig. 5:**
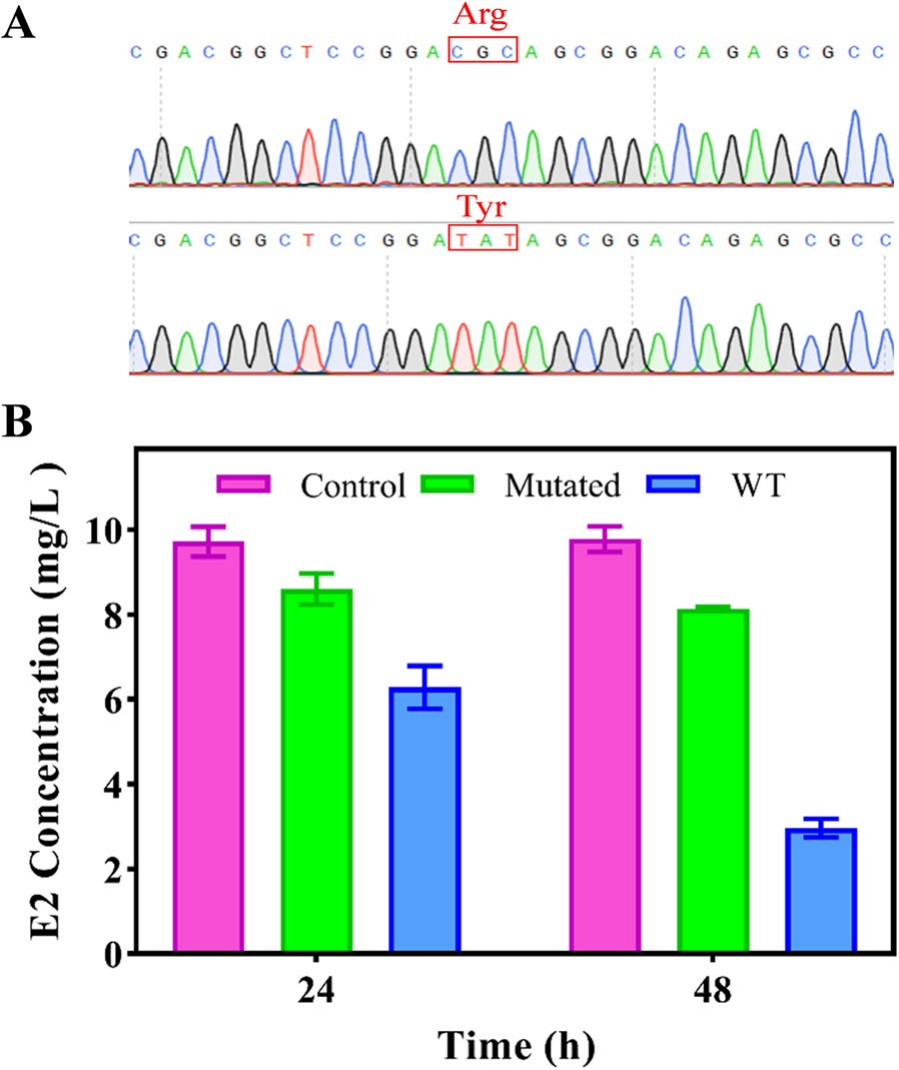
DNA sequencing validation of mutant proteins and comparison of E2 degradation efficiency

To further verify whether amino acid residue ARG215 is a key amino acid for the E2 degradation by 17β-HSD-0095, ARG215 (arginine) was mutated to Tyr (tyrosine) (Fig. 5A). Then, the degradation ability of wild-type protein and mutant protein for E2 was compared, and it was found that the degradation ability of the mutant protein for E2 was significantly weakened after Tyr substitution for Arg (Fig. 5B). The E2 degradation ability of the mutant protein with the same treatment was reduced by 51.69% compared to the wild-type protein.

### Remediation of wastewater E2 pollution by 17β-HSD-0095

**Fig. 6:**
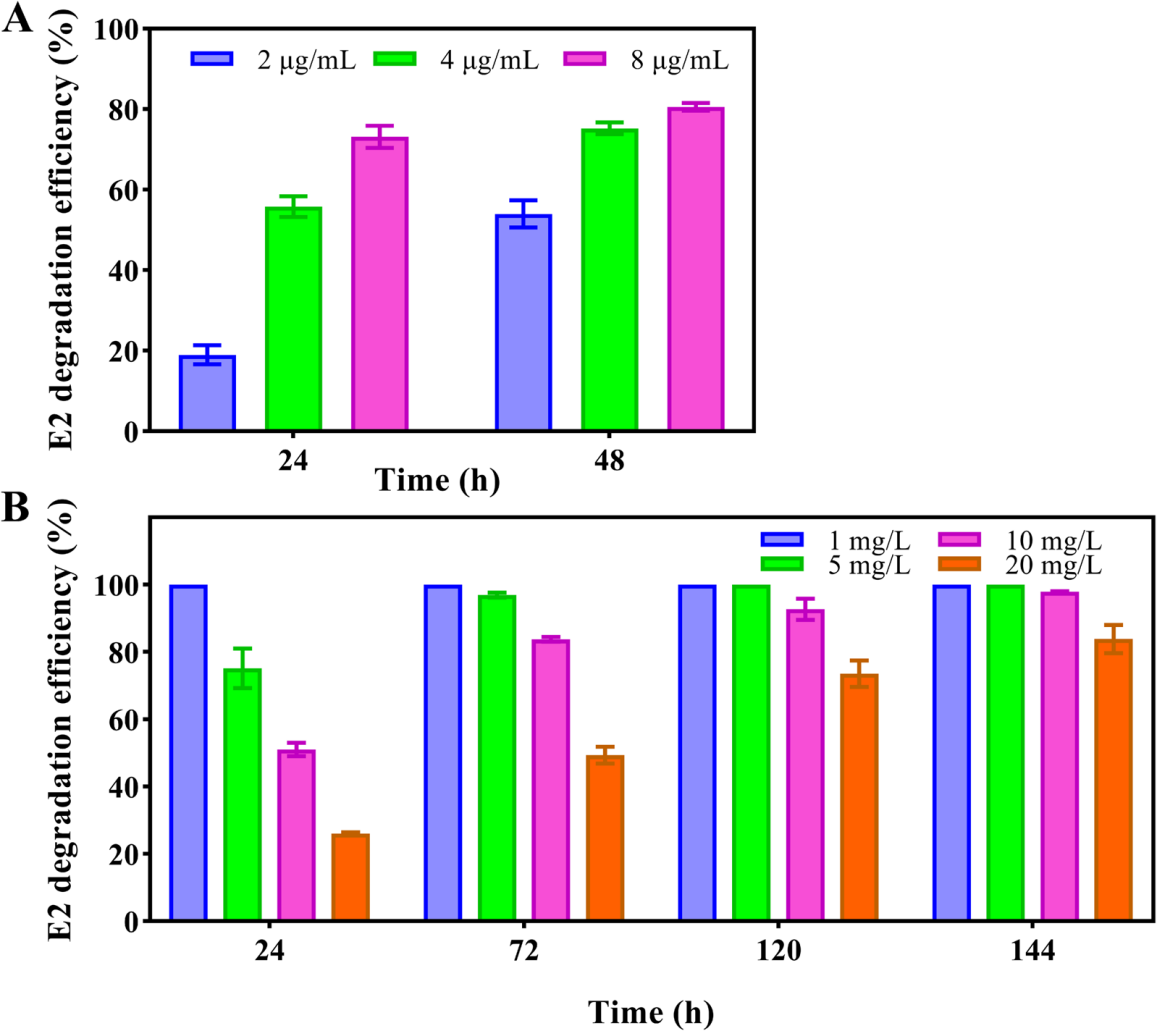
E2 degradation by 17β-HSD-0095. **A** E2 degradation (10 mg/L) in MSM. **B** E2 degradation in synthetic livestock wastewater by 4 μg/mL protein

In MSM, the degradation efficiency of E2 by 17β-HSD-0095 increased with the increase in enzyme inoculum and time (Fig. 6A). The degradation efficiency was the highest when the concentration of 17β-HSD-0095 was 8 μg/mL; under this condition, 73.15% of E2 was degraded at 24 h and 80.56% of E2 was degraded at 48 h. It is important to note that during 48 h, the degradation rate of E2 reached 75.23% when the enzyme concentration was 4 μg/mL. The difference in E2 degradation efficiency between 4 μg/mL and 8 μg/mL of enzyme was only 5.33%. Secondly, considering the cost factor, the final concentration of the enzyme was selected as 4 μg/mL for subsequent experiments.

The results of E2 degradation in synthetic livestock wastewater by 17β-HSD-0095 protein showed that the degradation protein could effectively control E2 contamination (Fig. 6B). 17β-HSD-0095 could completely remove 1 mg/L of E2 within 24 h while 5 mg/L of E2 was completely degraded within 120 h. For higher concentrations of E2, like 10 mg/L and 20 mg/L, they could also be degraded 97.87% and 83.78%, respectively, during incubation.

## Discussion

Estrogenic residues in the environment threaten the health of humans, animals and plants and affect the development of living organisms [1]. Therefore, it is urgent to control estrogen pollution. The SDR superfamily is widespread, which is active for a wide range of substrates including SEs [27], so it can be used to remove residual estrogen E2 in the environment. However, reports on E2 degradation by SDRs are still limited, especially studies on E2 removal from wastewater. In a prior investigation, it was discovered that E2 induction increase the expression of 17-HSD-0095 in the strain MZT7, and heterologously expressed recombinant cells had the capacity to degrade E2 [34]. In this study, the 17β-HSD-0095 protein was purified and its enzymatic properties were investigated. The degradation enzyme was used to remediate E2 contamination in farm wastewater, and the possible degradation mechanisms were explored by structural prediction and molecular docking analysis.

The amino acid sequence of 17β-HSD-0095 possesses the characteristic motifs of SDR family members (Gly-X-X-X-Gly-X-Gly and Tyr-X-X-X-Lys) as previously reported (Fig. 1) [29, 35, 36]. Numerous studies have shown that they are essential for the enzyme activity [30]. Then, the effects of temperature (10–70 °C) and pH (5–11) on the enzyme activity were determined. 17β-HSD-0095 appeared to have higher temperature tolerance and could efficiently convert E2 at a temperature of 20–50 °C, while the optimal reaction temperature was 40 °C (Fig. 3A). In contrast, 17β-HSDx purified from *Rhodococcus* sp. P14 was sensitive to high temperature, with an optimum temperature of 30 °C [36]. Consistent with previous studies, 17β-HSD-0095 also showed higher enzymatic activity under alkaline conditions [23, 36], and the optimal pH was 9 (Fig. 3B). In addition, the effects of different metal ions on enzyme activity were explored. The results showed that these ions either inhibited or had no effect on the enzymatic activity, indicating that the enzyme reaction was not dependent on the metal cofactor (Fig. 3C). In addition, the results of enzyme kinetic parameters indicated that 17β-HSD-0095 had a high binding capacity and catalytic reaction rate for E2 (Fig. 3D).

To further explore the mechanism of estrogen E2 degradation by 17β-HSD-0095 protein, we performed homology modeling and molecular docking analysis (Fig. 4). There is an evidence that homology modeling and molecular docking techniques can elucidate the mechanism of enzymatic degradation of contaminants, although the major areas of their application are in medicine and biology [33, 37, 38]. The optimal binding site between the enzyme and substrate molecule can be predicted by molecular docking. Xing et al. predicted that the VAL188 residue of CgSDR may play a key role in the patulin degradation [33]. Similarly, Zhao et al. analyzed the homology model and predicted that possible residues would affect the catalytic activity of degrading enzymes [39]. In the present study, we predicted that the ARG215 residue might affect the degradation of 17-HSD-0095 on E2. ARG215 formed a 1.8 Å hydrogen bond with the oxygen atom of E2, which was exactly at the C17 position of E2 (Fig. 4C). The role of hydrogen bonding is to maintain the stability of the substrate and enzyme interaction [38]. Moreover, the results at this point do not yet strongly suggest that the ARG215 residue plays an important role in the E2 biodegradation. For this reason, the ARG215 residue was targeted for mutation to Tyr (Fig. 5A). By comparing the degradation ability of the wild-type protein with that of the mutant protein for E2, we have found that the degradation ability of the mutant protein was significantly reduced (Fig. 5B). The above results suggest that the ARG215 residue has an important role in the E2 degradation, and its formation of hydrogen bonds with the oxygen atom of E2 may stabilize the active center of the degradase [33]. The degradation of 2,3-dichlorophenol by the degradation enzyme was reduced by 86% after the point mutation of 448-glycine residue to cysteine as reported by Fang et al. [37]. In another investigation, researchers had replaced 188-valine residue with alanine based on molecular docking results, and the degradation enzyme has been shown that has almost lost its ability to degrade patulin. Furthermore, in the study of glutathione-s-transferase degradation of chlorimuron-ethyl, mutations in key amino acids reduced the relative activity of the protein [40].

In comparison to microorganisms, enzymes are specific and effective biocatalysts that have more potential for biodegradation and elimination of environmental contaminants [36]. A 5 mg/L of E2 can be completely degraded by 17-HSD-0095 within 5 d, and higher concentrations of E2 can also be efficiently degraded, such that the concentration is already much higher than the residual in the actual environment (Fig. 6). It shows its potential to be applied to remediate E2 contamination in wastewater. Most studies on enzymes related to estrogen degradation in the last decades are based on fungal laccase [2, 3, 41–43]. Estrogen contamination in the environment can be efficiently removed by laccase, but metabolite studies have shown that its catalytic estrogen byproducts are mainly oligomers such as dimers and trimers [41, 42]. So it is necessary to develop enzymes that can completely break down estrogens into smaller molecules. Therefore, purification of bacterially encoded estrogen-degrading enzymes is of great significance. Although hydroxyl dehydrogenases encoded by bacteria have also been reported, such as OecA [44], 17β-HSDx [36], 3-oxoacyl-(acyl-Carrier-protein) reductase [23] and hsd17b14 [7], but their estrogen repair capacity in wastewater has not yet been investigated to the best of our knowledge.

In addition, enzymes play an important role in the decomposition of pollutants, but they cannot completely remove pollutants, because specific enzymes can only convert complex pollutants into simpler substances [45]. Therefore, it is necessary to combine other pollutant treatment methods, or a combination of multiple enzymes to completely remove the pollutants. Many functional enzyme reactions requires the participation of coenzymes, which can limit their application in wastewater, such as the NAD^+^ requirement for 17β-HSD-0095 protein in this study. To overcome this NAD^+^ requirement, in future studies we can construct recombinant bacteria containing co-expression plasmids, like the one constructed by Dong et al. [46] containing AzoRed2 and BsGDH, which can efficiently degrade azo dyes without the addition of coenzymes. Additionally, the free enzyme has limited catalytic activity and reusability [45]. The degrading enzyme will be immobilized in the following stage, which will boost the enzyme’s stability and activity and will improve the effectiveness of the pollutant removal process. [3, 45, 47]. In future, the molecular structure of 17β-HSD-0095 protein needs to be explored in depth and modified to elucidate its mechanism of action and enhance its environmental adaptability and E2 degradation ability. In addition, the degradation enzyme needs to be applied to actual wastewater such as swine wastewater, poultry wastewater and dairy wastewater to investigate its remediation ability.

## Conclusion

In this study, 17β-estradiol-degrading enzyme from *M. resistens* MZT7 was purified and characterized. The enzyme was expressed in a soluble form, which exhibited higher enzymatic activity under alkaline conditions (optimum pH 9). 17β-HSD-0095 has shown great potential for remediation of E2 pollution in synthetic livestock wastewater. Based on molecular docking and point mutation, the results have shown that the ARG215 residue of 17β-HSD-0095 protein play an important role in the E2 degradation. These findings provides valuable informations for exploring the mechanism of enzymatic E2 degradation and enzymatic remediation of estrogenic contamination in wastewater. In future, we plan to purify more degradation enzymes and will use them in combination for complete removal of estrogen contamination from the environment. Meanwhile, we will further analyze the structure of 17β-HSD-0095 and will perform molecular modification.

## Materials and methods

### Strains and chemicals

The *E. coli* BL21 (DE3) cells containing recombinant plasmid (pET-0095) were already preserved in our lab [34]. The strains were cultured in Luria–Bertani (LB) medium. Degradation and relative enzyme activity experiments were performed using Mineral Salt Medium (MSM). LB and MSM were prepared as previously described [34]. E2 (> 99% purity) was purchased from Solarbio Scientific Co., Ltd. (Beijing, China), nicotinamide adenine dinucleotide trihydrate (NAD^+^) from Sangon Biotech Co., Ltd. (Shanghai, China) and acetonitrile and methanol of HPLC quality were supplied by Thermo Fisher Scientific Co., Ltd (Shanghai, China).

### Amino acid sequence data analysis

17β-HSD amino acid sequences of other bacteria were obtained from GenBank and 17β-HSD-0095 sequence was repeatedly compared with other sequences using the Clustal W alignment method. Conserved binding domains of nucleotide sequences were compared using ESPript 3 (https://espript.ibcp.fr/ESPript/cgi-bin/ESPript.cgi) [48, 49].

### Purification of the E2 degrading enzyme

The genetically engineered strain was cultured in 1 L fresh LB medium containing 50 μg/mL kanamycin to an OD_600_ of about 0.5, at 37 °C. Then, a 0.3 mM IPTG was used to induce recombinant protein expression. After overnight incubation at 16 °C, cells were harvested by centrifugation at 10,000 rpm for 10 min. Next, the cells were resuspended in lysis buffer (50 mM NaH_2_PO_4_, 300 mM NaCl, 10 mM imidazole, pH 8.0) with a final concentration of 1 mM of phenylmethyl sulfonyl fluoride (PMSF), followed by disruption on ice with a sonicator for 30 min (the sonication work and gap times were 4 and 5 s). The cell fragmentation solution, supernatant, and precipitate were collected for SDS-PAGE to determine the type of protein expression. The recombinant bacteria produced without IPTG induction were used as controls.

The cell supernatants were mounted on Ni NAT Beads 6FF (Smart-Lifesciences Biotechnology Co., Ltd., Changzhou, China) affinity chromatography. Next, heteroproteins were removed using wash buffer (50 mM NaH_2_PO_4_, 300 mM NaCl, 20 mM imidazole, pH 8.0). Finally, the recombinant proteins were eluted by elution buffer (50 mM NaH_2_PO_4_, 300 mM NaCl, 250 mM imidazole, pH 8.0). Protein expression was evaluated using sodium dodecyl sulfate polyacrylamide gel electrophoresis (SDS-PAGE), and gels were stained with Coomassie brilliant blue R-250. The protein was dialyzed for 12 h via a dialysis bag (14 KD, 77 mm width, Sangon Biotech Co., Ltd., Shanghai, China) to remove salt ions and imidazole. The BCA protein assay kit (Takara, Dalian, China) was used to detect the protein concentration.

### Enzyme characterization

The generation of E1 was measured by HPLC to determine the enzymatic activity of 17-HSD-0095. The total reaction system was 1000 μL, including 900 μL MSM with E2 (final concentration 10 mg/L), 50 μL NAD^+^ (final concentration 500 μM) and 50 μL enzyme solution. The reaction mixture was reacted at 30 °C for 30 min, and then the reaction was terminated by heating in a boiling water bath for 10 min. As a blank control, the reaction system without enzyme addition was employed. The effects of temperature, pH, and different metal ions on the activity of 17β-HSD-0095 were determined. The enzyme activity at the optimal temperature, pH and without the addition of metal ions was set to 100%, and this was used to calculate the relative enzymatic activity under other conditions. The kinetic parameters (*Km* and *Vmax*) of 17-HSD-0095 at different E2 concentrations (0.018–0.40 mM) were calculated at pH 7.

### Homology modeling and molecular docking

The SWISS-MODEL web portal (https://swissmodel.expasy.org/) was used for homology modeling of 17β-HSD-0095 protien [37, 50]. The crystal structure of short-chain dehydrogenase from *Mycobacterium smegmatis* (Protein Date Bank: 8CXA_A) was selected as template. The quality of the models was assessed according to the Ramachandran plot [51]. The 3D structures of the small molecule ligands were obtained from the PubChem database (E2: CID5757). Ligand, receptor preprocessing and molecular docking were performed using AutoDock 4.2 to simulate the binding pattern of E2 to 17β-HSD-0095 upon NAD^+^ action. The results were analyzed and visualized in AutoDockTools and PyMOL.

### Point mutation

Modification of ARG215 residues by point mutation was based on molecular docking data. The recombinant plasmid pET-28a-0095 was used as a template for amplification mutagenesis using overlapping primers (F: TCCGGATATAGCGGACAGAGCGCCGGACCGGG; R: TGTCCGCTATATCCGGAGCCGTCGGTCATCCC). The amplification products were then digested using DNA methyltransferase and transformed into receptor cells (*E. coli* DH5α). Positive clones were picked and sequenced for verification. The plasmid was then transformed into *E. coli* BL21 (DE3) for protein expression. The degradation ability of the wild-type (WT) protein and the mutated protein to E2 was compared according to the above method.

### Wastewater remediation tests

To evaluate the performance of degradation enzymes in synthetic livestock wastewater. First, we conducted experiments on MSM to identify the ideal level of enzyme addition for degradation. In a 25 mL conical flasks with a total system of 10 mL, 10 mg/L of E2, 500 μM NAD^+^, and different final concentrations (2 μg/mL, 4 μg/mL and 8 μg/mL) of 17-HSD-0095 protein were put to carry out degradation tests. Three repetitions of each therapy were given under the identical circumstances, with the treatment without protein serving as control. The flasks were incubated at 30 °C in a dark environment at 130 rpm in a shaking incubator. Samples were taken out at the designated time points to determine the residual amount of E2 and degradation efficiency. Next, synthetic livestock wastewater was prepared according to the method described by Tang et al. [52]. Degradation tests were performed by adding different concentrations of E2 and concentrations of 4 μg/mL of protein to the wastewater. The degradation system, NAD^+^ level and incubation settings were kept constant. Each treatment was repeated three times and E2 residues were detected at the designated times.

### Detection method

The extraction and HPLC (Shimadzu LC-2030 plus, Japan) detection of E2 in the degradation system was performed as previously described with minor modifications [34]. Briefly, an equal volume of methanol was added to the degradation system, the mixture was dissolved by ultrasonication and centrifuged at 10,000 rpm for 5 min, and the supernatant was filtered through a 0.22 μm filter and detected by HPLC/UV for estrogen concentration.

## Data Availability

All data generated or analyzed during this study are included in this article.
